# Changes in MELD-Na & Fibroscan Scores in Patients with Chronic Hepatitis-B on Entecavir: A Cohort Analysis

**DOI:** 10.12669/pjms.42.3.12534

**Published:** 2026-03

**Authors:** Bader Faiyaz Zuberi, Faiza Sadaqat Ali, Tazeen Rasheed, Nimrah Bader

**Affiliations:** 1 Bader Faiyaz Zuberi, FCPS. Meritorious Professor, OMI Hospital, Karachi, Sindh, Pakistan; 2 Faiza Sadaqat Ali, FCPS Senior Registrar, Department of Medicine/Gastroenterology, Dow University of Health Sciences, Karachi, Sindh, Pakistan; 3 Tazeen Rasheed, FCPS Associate Professor, Department of Medicine/Gastroenterology, Dow University of Health Sciences, Karachi, Sindh, Pakistan; 4 Nimrah Bader, MD Assistant Professor, Department of Rheumatology, University of Oklahoma Health Sciences Center, Oklahoma, OK, USA

**Keywords:** Hepatitis-B, MELD-Na, Chronic Liver Disease, Fibroscan

## Abstract

**Objective:**

To determine on-treatment changes in MELD-Na & Fibroscan scores after a period of six months in patients with chronic Hepatitis-B who are treated with Entecavir (ETV).

**Methodology:**

This observational cohort was conducted at Department of Medicine/Gastroenterology, Dow Medical College affiliated with Dow University of Health Sciences, Karachi, Pakistan during the period of August 2019 to December 2024. Patients of either gender aged between 18 - 80 years attending hepatitis clinic and who are on treatment for Hepatitis-B with Entecavir were inducted after informed consent. MELD-Na & Fibroscan scores were recorded at induction and after six months of treatment with ETV.

**Results:**

A total of 288 patients were enrolled including 153 (53.1%) were males with mean age of 37.6 ±11.6 years and 135 (46.9%) were females with mean age of 43.5 ±9.6 years. The difference in age between genders was significant (*p* < .001). Comparison of parameters at induction of study and after six months of treatment with Entecavir using Paired Sample T-Test, we found significant improvement in all parameters except serum albumin which showed significant reduction in levels after six months. MELD-Na Score improved from 9.38 to 8.94 (*p* <.001). CTP Score & Fibroscan improved from 5.7 to 5.66 (*p* <.001) & 8.95 to 8.61 (*p* <.001) respectively. Albumen values reduced from 4.08 to 3.98 (*p* <.001).

**Conclusion:**

Significant improvement in MELD-Na & Fibroscan scores were observed in patients on treatment with ETV.

## INTRODUCTION

The Model for End-Stage Liver Disease (MELD) score is a validated scale used for determining the severity of chronic liver disease.^1^ It utilizes serum bilirubin, International Normalized Ratio (INR) and serum creatinine. It has proven to be useful in an increasingly number of varied clinical situations.^2^ MELD score was initially formulated to predict survival among patients of decompensated cirrhosis undergoing transjugular intrahepatic portosystemic shunt but was later on found to be a valid predictor of mortality among patients with end-stage liver disease.^3^ MELD score is also used to predict survival in patients of cirrhosis having complications like infections, upper gastrointestinal bleeding, in patients of fulminant hepatic failure and alcoholic hepatitis.^4^,^5^ In patients of cirrhosis, increasing MELD is associated with an increase in three-month mortality risk.^3^

Antiviral therapy alters the natural course of chronic Hepatitis-B (CHB) related cirrhosis as is determined by improvement in MELD score over time. On-treatment improvement of MELD scores has found to be associated with decreased risk of hepatic complications and mortality among patients with CHB. In a study conducted by Yip TC et al reported that among cirrhotic CHB patients treated with Entecavir (ETV) or tenofovir for six months the mean MELD score decreased from 12.3 ±5.5 at baseline to 11.0 ±4.7.^6^

Hyponatremia correlates with unfavorable complications of cirrhosis like ascites and hepatorenal syndrome. To additional augment the MELD-based liver allocation system, serum sodium (Na) was incorporated into MELD score and two mathematical equation including MELD score and Na were developed and named as MELD-Na and MELDNa. The MELD-Na improved the mortality predictions and reportedly avoid 7% of deaths on the wait list.^7^ Comparisons of all three showed that MELD-Na and MELDNa, both have better predictive value as compared to MELD.^8^,^9^

This observational cohort study was designed to determine the change in MELD-Na & Fibroscan scores over a period of six months in CHB patients who are being treated with ETV. This would help the patients studied in better monitoring and disease course prediction and better management of complications. The study would also create general awareness among doctors who are treating HBV, the need to monitor patients under treatment. The objective was to determine on-treatment changes in MELD-Na score after a period of six months in patients with chronic Hepatitis-B who are taking ETV.

## METHODOLOGY

This observational cohort was conducted at Department of Medicine/Gastroenterology, Dow Medical College affiliated with Dow University of Health Sciences, Karachi, Pakistan during the period of August 2019 and December 2024. Sample size calculation was done using reported change in 11% of HBV patients on treatment.^6^ A sample size of 94 achieves 90.1% power to detect a difference (P1-P0) of 0.15 using a two-sided exact test with a significance level (alpha) of 0.050. Sample size calculation was done using PASS version 19.0.9 software.

### Ethical Approval:

Written informed consent was obtained from each patient included in the study. This study was approved by the DUHS Intuitional Review Board (IRB) ethics committee. Approval No.: IRB-1336/DUHS/Approval2019/508, Date: August 1, 2019. All participants provided written informed consent before any study procedures and agreed to receive interventions.

### Inclusion & Exclusion Criteria:

All patients of either gender aged between 18 - 80 years attending hepatitis clinic and who are on treatment for Hepatitis-B with ETV were inducted by non-probability convenience sampling. Patients with CHB with Child’s Class ‘C’ at the time of presentation, co-infection with hepatitis C and/or D, suffering from concomitant diseases, e.g., autoimmune liver disease, metabolic liver diseases like hemochromatosis, Wilsons’s disease or hepatocellular carcinoma at the time of presentation were excluded. Patients who discontinued treatment for any reason during the study period, were also excluded.

### Data Collection Procedure:

Data was collected by the authors and study proformas were filled. Written informed consent was taken. History was taken for demographic variables like age, gender, contact numbers, duration of treatment, and was recorded in the proforma. The presence of ascites, hepatic encephalopathy and history of renal dialysis were also recorded. Abdominal ultrasound and Fibroscan were done by expert radiologist. Blood samples were withdrawn by trained phlebotomist for total bilirubin, serum sodium, INR, serum creatinine and albumin at time of induction into study. Tests were run at the hospital’s central laboratory. MELD-Na and CTP Scores were calculated.

### Follow up:

Patients were followed up for six months while on treatment. A reminder phone call was given to patients by secretary of primary investigator at the completion of six months. Clinical examination and all previous tests were repeated and noted in study proforma. MELD-Na and CTP Scores were re-calculated.

### Data Analysis Procedure:

Means ±SD age, bilirubin, creatinine, INR, serum sodium, serum albumin, fibroscan values, MELD-Na & CTP Scores were obtained and compared using Paired Sample T-Test. Frequencies and percentages of gender, ascites and hepatic encephalopathy were reported. MELD-Na scores of month 0 & 6 were recoded into new variables on basis of their score values of <10 and ≥10. Frequencies of these new variables were calculated and compared by χ^2^-test. P value ≤.05 was used as significant. Statistical analysis was done using SPSS version 26.0 (SPSS, Inc., Chicago, Illinois).

## RESULTS

During study period 288 patients were enrolled, 153 (53.1%) were males with mean age of 37.6 ±11.6 years and 135 (46.9%) were females with mean age of 43.5 ±9.6 years. The difference in age between genders was significant (*p* < .001). Mean serum sodium levels were significantly lower in females (*p* <.001) and CTP scores were also significantly lower in females (*p* <.001). Rest of the parameters of study did not show any significant difference in gender on Student’s t-test. Details are given in Table-I. In comparison of parameters at induction of study and after six months of treatment with ETV using Paired Sample T-Test, T Details mentioned in Table-II.

**Table-I:** Age, creatinine, INR, Sodium, albumin, MELD-Na, CP Score and Fibroscan Values Compared in Gender by Student’s T-test.

	Gender				
	Male		Female		
	Mean	SD	Mean	SD	Sig
Age	37.63	11.70	43.47	9.70	<.001*
Serum creatinine	.96	.32	.89	.27	.057
INR	1.22	.15	1.30	.60	.108
Serum Sodium	138.12	2.55	136.84	3.69	<.001*
Serum Albumin	4.01	.83	4.17	.73	.079
MELD-Na	9.53	2.06	9.22	1.91	.189
CTP Score	5.90	1.37	5.47	.79	.001*
Fibroscan	8.71	4.12	9.21	2.32	.213

* Significance ≤ .05.

**Table-II:** Mean MELD-Na, CTP, Fibro scan scores, serum bilirubin, creatinine, INR, Sodium and albumin at baseline and after six months of treatment with Entecavir.

		Mean	SD	p-value
1	MELD-Na Month 0	9.38	1.99	<.001*
	MELD-Na Month 6	8.94	1.74	
2	CTP Score Month 0	5.70	1.15	<.001*
	CTP Score Month 6	5.66	1.07	
3	Fibroscan Month 0	8.95	3.39	<.001*
	Fibroscan Month 6	8.61	3.29	
4	Bilirubin Month 0	1.21	.69	<.001*
	Bilirubin Month 6	1.02	.48	
5	Serum creatinine Month 0	.93	.30	<.001*
	Serum creatinine Month 6	.85	.21	
6	INR Month 0	1.26	.42	<.001*
	INR Month 6	1.24	.24	
7	Serum Sodium Month 0	137.52	3.19	<.001*
	Serum Sodium Month 6	141.82	4.26	
8	Serum Albumin Month 0	4.08	.78	<.001*
	Serum Albumin Month 6	3.98	.70

* Significance ≤.05.

## DISCUSSION

The presented results demonstrate notable trends in the biochemical and prognostic measurements for patients undergoing ETV therapy for Chronic Hepatitis-B (CHB). Significant observations from the study include changes in serum albumin levels, MELD-Na scores, and other associated markers of liver function.

### Biochemical Progress:

Serum albumin levels showcased a decrease over the six-month study period, with levels reducing from 4.08 ± 0.78 g/dL at baseline to 3.98 ± 0.70 g/dL after six months of treatment. Although the reduction was statistically significant (p < .001), it suggests potential nuanced effects of ETV therapy on protein synthesis within the liver. This observation highlights the need for further research to delineate whether the decrease is directly related to ETV therapy or influenced by underlying chronic liver disease (CLD) and other patient variables such as nutritional status.^10^,^11^

### Prognostic Insights: MELD-Na Scores:

MELD-Na scores improved significantly during the study period, decreasing from 9.38 ± 1.99 to 8.94 ± 1.74 (*p* <.001), indicating enhanced liver functionality and reduced severity of liver disease within the cohort.^12^ These findings align with previous studies, such as Yip TC et al., where MELD scores also decreased after six months of ETV therapy.^6^ The comparative analysis adds to the growing evidence of the clinical utility of MELD-Na scores as a reliable predictive measure for liver dysfunction improvement under ETV therapy.

### Comparative Parameters: INR and Serum Sodium:

Unlike MELD-Na improvements, other biochemical parameters showed varying dynamics. INR levels experienced a significant improvement from 1.26 ± 0.42 to 1.24 ± 0.24 (*p* < .001), while serum sodium levels demonstrated a significant increase from 137.52 ± 3.19 mmol/L to 141.82 ± 4.26 mmol/L (*p* < .001). These observations potentially reflect the interplay between dietary habits, inherent status of CLD, and treatment effects, emphasizing the multifactorial nature of liver disease outcomes. Comparatively, Yip TC et al^6^ documented similar trends in these markers, further validating the findings but also underscoring the need for personalized management approaches.

### Clinical Implications:

ETV therapy emerges as a promising intervention for managing CHB, particularly in improving predictive measures such as MELD-Na scores. The significant progress documented in liver status provides compelling evidence for the role of nucleos(t)ide analogues in enhancing clinical outcomes. However, the non-significant trends observed in other biochemical markers, coupled with the reduction in serum albumin levels, warrant cautious interpretation. These results highlight the complexity of liver disease management and the need for comprehensive treatment strategies addressing biochemical, dietary, and clinical factors.

Major development in treatment of Chronic Hepatitis-B (CHB) occurred with development of oral antiviral treatment with nucleos(t)ide analogs (NUA).^13^ NUAs have shown to effectively suppress Hepatitis-B virus (HBV) replication, improving histology and decreasing incidence of hepatocellular carcinoma in HBV patients.^14^ Treatment with ETV have demonstrated biopsy proven long-term fibrosis regression in addition to biochemical and virological progresses.^15^ Model for end-stage liver disease (MELD) score, was developed to predict survival after trans jugular intrahepatic portosystemic shunt,^16^ but was also found to accurately predict mortality in patients with cirrhosis. Currently it is one of the important criteria for prioritization of patients for liver transplant.^17^ It has been documented to predict survival in many liver diseases, e.g., variceal bleeding,^18^ hepatorenal syndrome,^2^ and spontaneous bacterial peritonitis.^19^

We used MELD-Na scores to non-invasively document the changes in liver status in patients on treatment with ETV. In our cohort we found significant improvement in MELD-Na scores in these patients. MELD-Na improved from 9.38 ±1.99 to 8.94 ±1.74 in six months of study period and it was statistically significant (*p* = .041). In a similar study by Yip TC et al, the mean MELD score decreased from 12.3 ± 5.5 to 11.0 ± 4.7 after six months of ETV therapy.^6^ They also demonstrated improvement in mean INR from 1.3 ± 0.3 to 1.2 ± 0.2 and the mean serum sodium increased from 138.7 ± 3.7 to 139.2 ± 3.6 mmol/L, while the mean serum creatinine increased from 1.1 ± 1.0 to 1.2 ± 1.1 mg/dL (paired t-test, *p* < 0.001). In our study there was non-significant increase in INR from 1.22 ±0.15 to 1.30 ±0.6 (paired t-test, *p* = .358) and non-significant decrease in serum sodium from 138.12 ±2.55 to 136.84 ±3.69 (paired t-test, *p* = .276). This could be due to status of CLD and dietary habits of the patients.

### Future Directions:

While the study successfully demonstrates the short-term benefits of ETV therapy in CHB patients, the relatively short duration of the research limits its ability to capture long-term sustainability of these improvements. Further investigations with larger cohorts, extended follow-up periods, and diverse patient demographics are essential to enhance the generalizability of these findings. Additionally, exploring the interplay between CLD status, dietary influences, and ETV effects on biochemical markers could provide critical insights for optimizing treatment protocols.

### Strength & Limitations:

Strength of this manuscript lies in its real-time cohort study design, which offers valuable insights into the progression and treatment outcomes of Chronic Hepatitis-B (CHB) using ETV therapy. Furthermore, the study adds to the growing body of research by comparing MELD-Na score changes and integrating biochemical markers over a six-month treatment period. However, there are limitations to consider. The study relies on a relatively short duration, which might not capture long-term changes or sustainability of improvements in liver function. In addition, the dietary habits and underlying clinical conditions of the patients might have influenced certain parameters such as serum sodium and INR, potentially limiting the generalizability of observations across diverse populations. Lastly, the sample size and specific demographic characteristics of the cohort may further constrain the broader applicability of the results.

## CONCLUSION

In summary, the study highlights the clinical utility of ETV therapy for CHB, particularly in improving predictive measures of liver dysfunction like MELD-Na scores. While significant progress in liver status was documented, limitations such as short duration and potential confounding factors-diet and underlying CLD-warrant further research. These findings contribute valuable real-world data to the growing body of evidence supporting the role of nucleos(t)ide analogue therapies in the management of Chronic Hepatitis-B, while underscoring the need for comprehensive and personalized treatment strategies to address the complexity of liver disease.

### Author’s Contributions:

**BFZ:** Conceptualized and designed the study.

**NB:** Data analysis, interpreted the findings. Provided medical and technical advice for the project

**FSA:** Drafted the original manuscript under supervision.

**TR & FSA:** Contributed to the communication with Hepatitis-B virus-positive individuals and organized their anonymous data.

**TR & NB:** Supported and supervised the statistical analysis.

**BFZ, FSA, TR & NB:** Participated in the discussion and critical review of the manuscript.

All authors contributed to revisions and approved the final manuscript and are responsible for integrity of data and study.
